# Supplementary figures and images for: Differential Impacts on Host Transcription by ROP and GRA Effectors from the Intracellular Parasite Toxoplasma gondii
Source: mBio. 2020 Jun 9;11(3):e00182-20. doi: 10.1128/mBio.00182-20 (PMC7373195; doi:10.1128/mBio.00182-20)

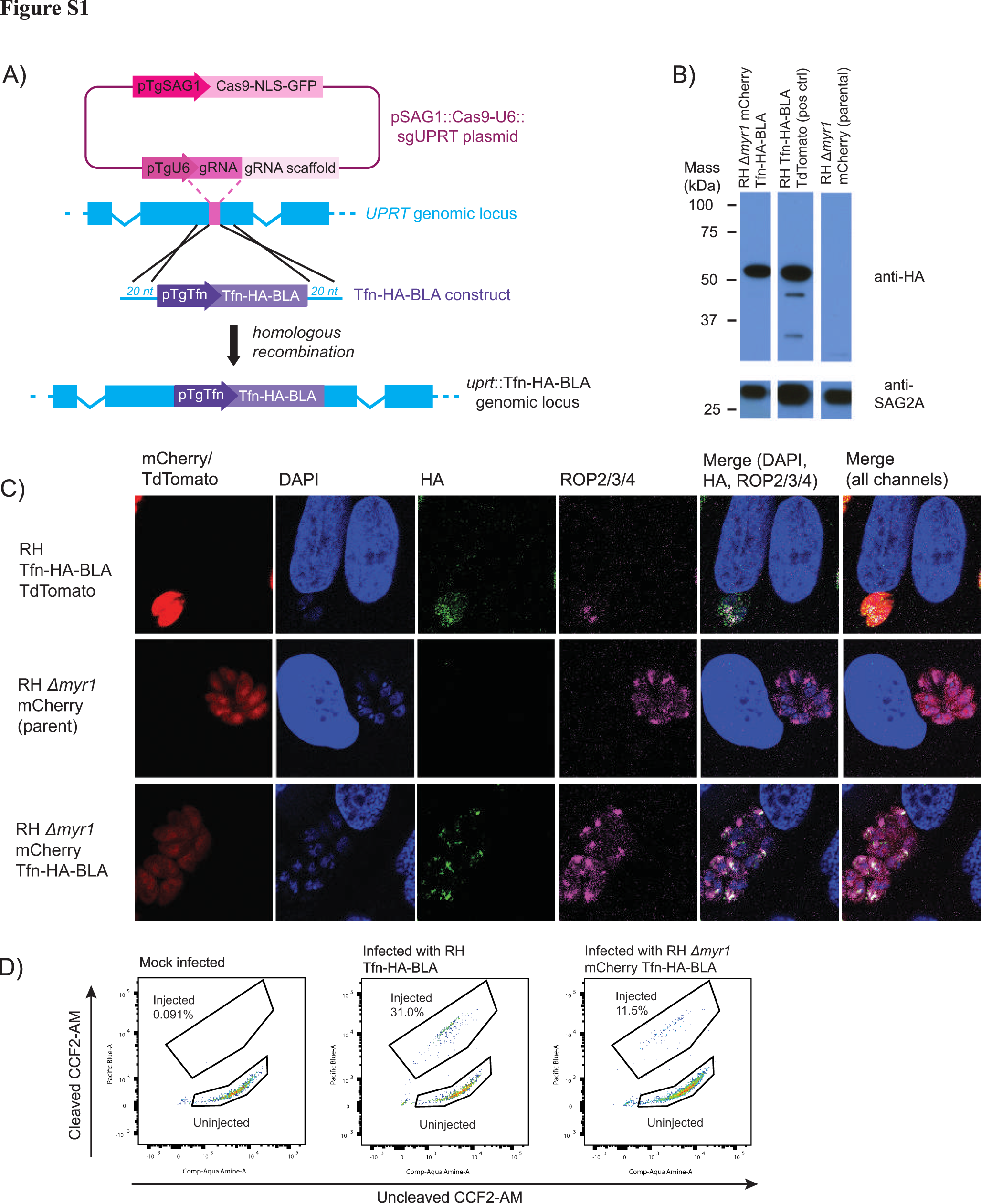

Supplement: FIG S1 [file mBio.00182-20-sf001.tif]

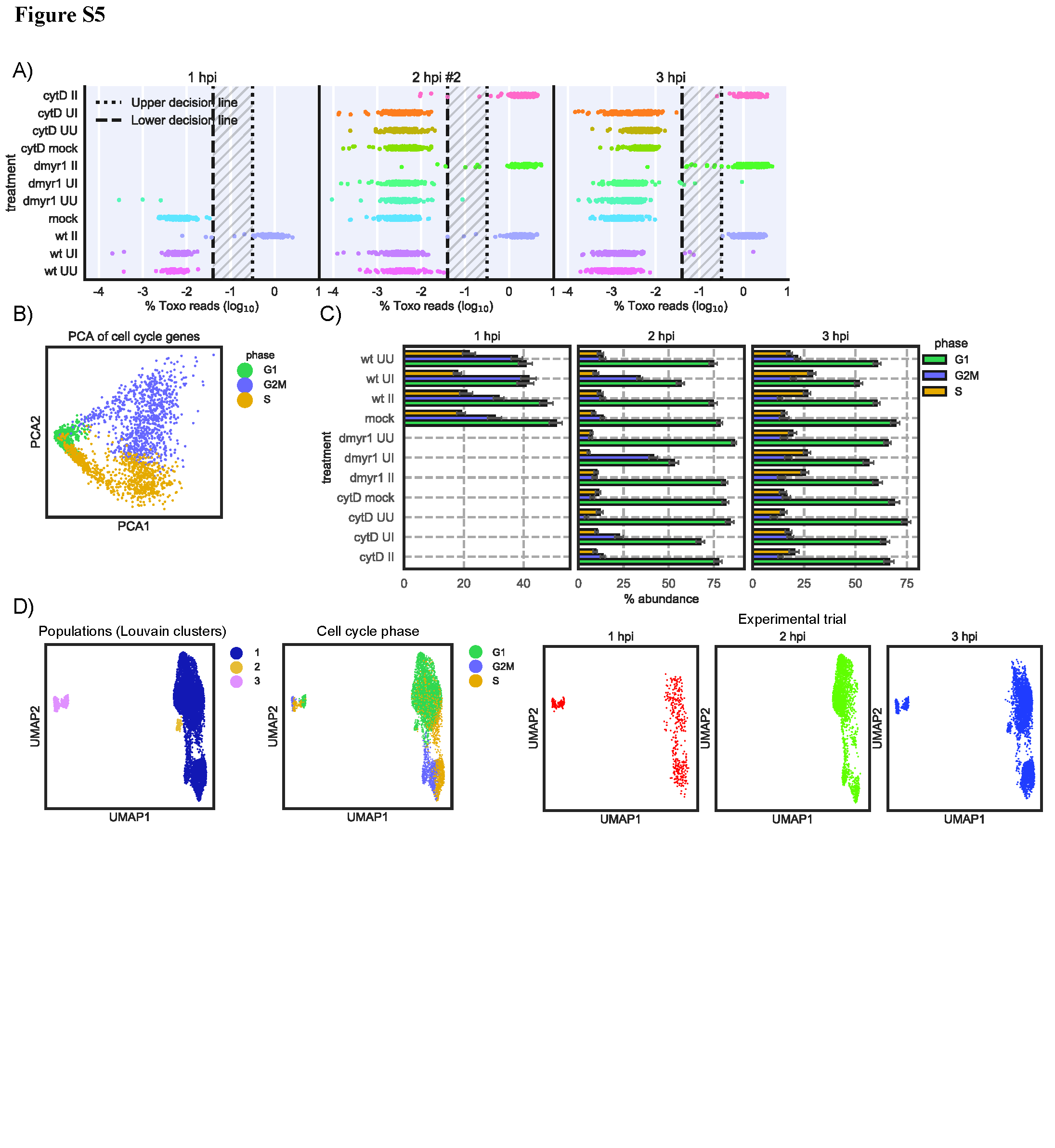

Supplement: FIG S5 [file mBio.00182-20-sf005.tif]

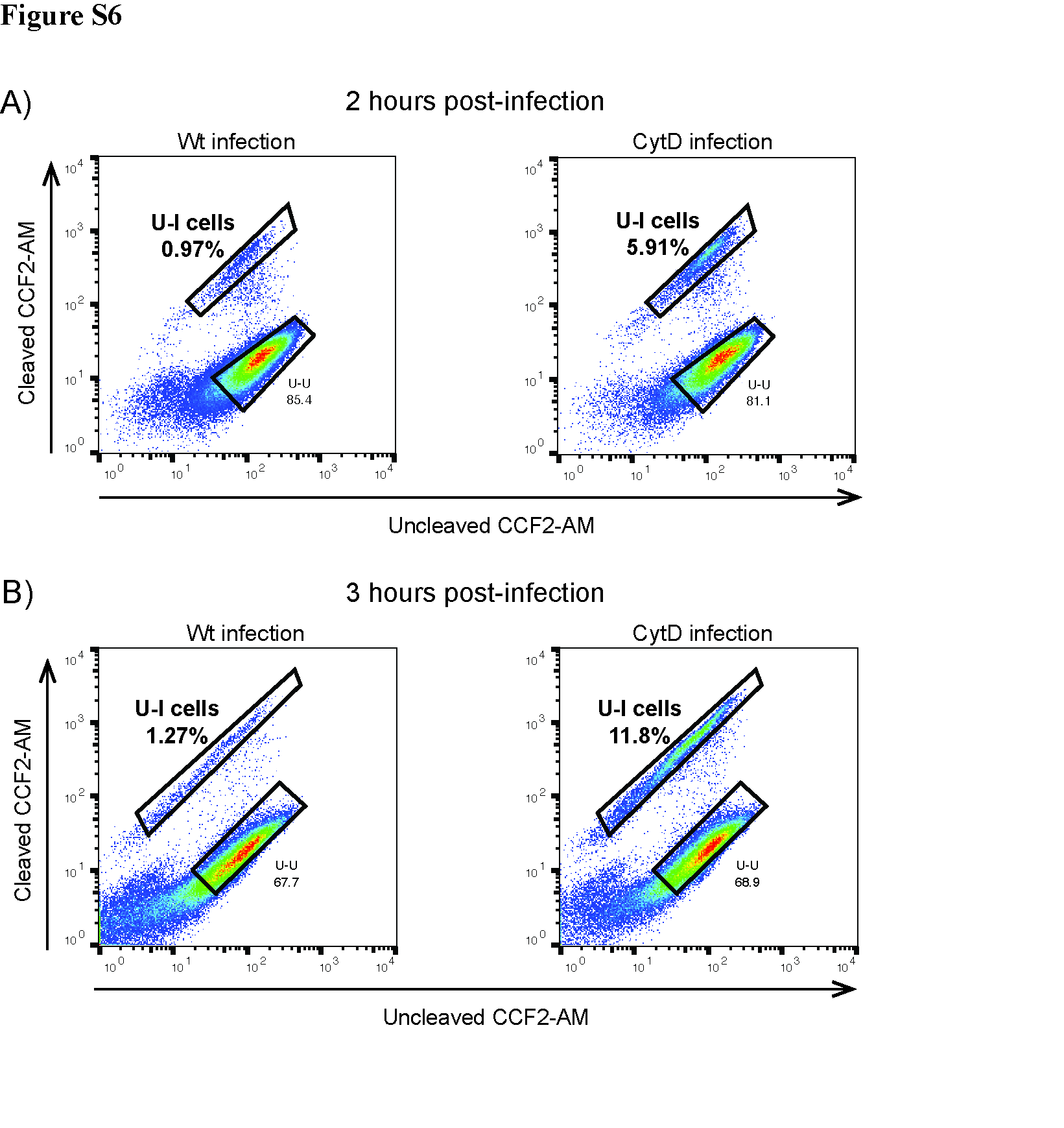

Supplement: FIG S6 [file mBio.00182-20-sf006.tif]
